# Variations in Soil Bacterial Composition and Diversity in Newly Formed Coastal Wetlands

**DOI:** 10.3389/fmicb.2018.03256

**Published:** 2019-01-09

**Authors:** Wenbing Li, Xiaofei Lv, Junchao Ruan, Miao Yu, Yao-Bin Song, Junbao Yu, Ming Dong

**Affiliations:** ^1^Key Laboratory of Hangzhou City for Ecosystem Protection and Restoration, College of Life and Environmental Sciences, Hangzhou Normal University, Hangzhou, China; ^2^College of Quality and Safety Engineering, China Jiliang University, Hangzhou, China; ^3^Key Laboratory of Coastal Environmental Processes and Ecological Remediation, Yantai Institute of Coastal Zone Research, Chinese Academy of Sciences, Yantai, China; ^4^Institute for Advanced Study of Coastal Ecology, Ludong University, Yantai, China

**Keywords:** coastal ecosystem, ecological replacement, microbial community, sediment, Yellow River Delta

## Abstract

Coastal ecosystems experience some of the most active land–ocean interactions in the world, and they are characterized by high primary productivity and biological diversity in the sediment. Given the roles of microorganisms in soil biogeochemical cycling and their multifaceted influence on soil ecosystems, it is critical to understand the variations and drivers of soil microbial communities across coastal ecosystems. Here, we studied soil bacterial community dynamics at different sites (from seawater to freshwater) in the Yellow River Delta, China. Bacterial community composition and diversity over four seasons were analyzed through 16S rRNA genes. Notably, the bacterial community near the ocean had the lowest alpha-diversity when compared with the other sites. No significant differences in bacterial communities among seasons were found, indicating that seasonal variation in temperature had little influence on bacterial community in the newly formed wetlands in the Yellow River Delta. Bacterial community structure changed substantially along the salinity gradient, revealing a clear ecological replacement along the gradual transformation gradient from freshwater to seawater environment. Redundancy analysis revealed that salinity was the main driver of variations in bacterial community structure and explained 17.5% of the variability. Our study provides a better understanding of spatiotemporally determined bacterial community dynamics in coastal ecosystems.

## Introduction

Coastal wetlands, including saltwater and freshwater wetlands within coastal watersheds, are the typical transition zones between land and ocean. They provide favorable habitat for a wide variety of terrestrial and aquatic organisms and play important ecological and economic roles (Lv et al., 2016). Despite their vast importance, extremely rapid degradation and loss of coastal wetlands still continue because of habitat destruction, hydrological alteration, climate change, overexploitation, and pollution (Lv et al., 2016). The global decline in coastal wetlands is affecting many critical benefits and ecosystem services provided by coastal ecosystems (Barbier et al., 2011; Lv et al., 2016). Therefore, more focused protection strategies are required to reverse this trend. Recently, much attention has been directed toward formulating and implementing sustainable management strategies for wetlands.

The Yellow River Delta (YRD) is a typical coastal wetland that has naturally formed because of tremendous sediment load from the Yellow River. Thus, it plays an important role in the globally integrated, riverine sediment supply (Milliman and Syvitski, 1992; Yi et al., 2015; Zhang et al., 2016). There are three major factors that affect the vulnerability of these mega-deltas: human activity, sea level rise, and climate change (Yi et al., 2015; Zhang et al., 2016). Understanding the linkages between naturally occurring deltaic processes, anthropogenic impacts, and the internal and external driving forces is critical (Syvitski et al., 2009; Nicholls and Cazenave, 2010; Yi et al., 2015).

Influenced by land–ocean interactions, newly formed natural wetlands in the YRD contain numerous zonal habitats in a small geographic area, with highly specific plant communities and coastal wetland vegetation belts distributed perpendicular to the Yellow River, from the sea to the land (Yu et al., 2012). Furthermore, special soil types have developed for each vegetation zone from the fluvial sediment because of vegetation, soil-water salinity, and topography. Therefore, the YRD is attractive to researchers in several fields: sedimentary evolution (Liu et al., 2009; Qiao et al., 2011; Yi et al., 2015), deltaic geomorphology (Xue, 1993; Saito et al., 2000), modern sedimentary process (Wiseman et al., 1986; Chu et al., 2006), and the effects of vegetation type on soil properties (Wang et al., 2006; Cui et al., 2009).

Microorganisms have important effects upon local ecological processes (e.g., Cyanobacteria have high rates of primary production), biogeochemical cycles (e.g., nitrogen and carbon transformations), and sustainability of coastal soil ecosystems (Yu et al., 2012; Lv et al., 2016; Ma et al., 2016). In addition, invertebrates, fish, and shorebirds living in the coastal ecosystems also prey on microbes as a natural source of food (Schimel et al., 2007; Lv et al., 2016). Owing to large spatial and seasonal variations in coastal ecosystems, it is a challenge to identify the drivers of microbial community composition and distribution in complex and varied environmental conditions. Coastal ecosystems play important ecological and economic roles; newly formed coastal wetlands are especially vulnerable. Microorganisms drive biogeochemical cycling and determine the sustainability of soil in coastal ecosystems. Coastal wetland ecosystems in the YRD have clearly a horizontal distribution of vegetation zones, along with changes in soil salinity from seaside to inland (Yu et al., 2015a). However, variation in microbial communities in newly formed coastal wetlands along a salinity gradient is poorly understood. High through-put molecular technologies provide an opportunity for the cultivation-independent analysis of microbial community composition and structure in coastal wetlands with large spatial and seasonal variations.

We hypothesize that there are spatial and seasonal changes in bacterial community structure and function in newly formed coastal wetlands in the YRD, reflecting systematic changes in site conditions along a salinity gradient. In this study, we used intensive field sampling of newly formed coastal wetlands in the YRD, to investigate spatial and seasonal variations in bacterial communities and the factors affecting that variability. Bacterial community was determined in newly formed wetlands by sequencing 16S rRNA gene amplicons with the Ion Torrent Personal Genome Machine (PGM) platform. We analyzed these data to reveal (1) spatial and seasonal microbial community changes in newly formed coastal wetlands and (2) the dominant driving factors for those changes.

## Materials and Methods

### Study Area

The current study was conducted in the YRD (118.6° E-119.3° E, 37.6° N-38.2° N) in the northern Shandong Province of China, an area with a temperate and semi-humid continental monsoon climate. The average temperature is 11.7–12.6°C and average annual precipitation is 530–630 mm, of which 70% is rainfall from June to September (in summer and fall); average annual evaporation is 1900–2400 mm (Li et al., 2015). The youngest and the most extensive newly formed wetland ecosystem in the warm temperate zone of China occurs in the YRD (Lv et al., 2016).

From 1976 to 2009, the net increase of delta shoreline length was approximately 61.64 km, with an annual increase of approximately 1.81 km; net extension of newly formed wetland area was approximately 309.81 km^2^ with a rate of approximately 9.11 km^2^ year^-1^ in the YRD (Yu et al., 2012, 2015a).

According to Food and Agriculture Organization (FAO) soil classification, the dominant soil types in the study area are Calcaric Fluvisols, Gleyic Solonchaks, and Salic Fluvisols (Yu et al., 2015a). 400 plant species have been recorded in the area, of which 55.1% are halophytes or halophiles such as *Tamarix chinensis*, *Suaeda salsa*, and *Phragmites australis*. In the coastal wetland, biological communities distribute zonally (i.e., from a community without vegetation to one with vegetation dominated by *S. salsa*, *T. chinensis*, *P. australis*, and *Typha orientalis*), along a descending salinity gradient. Wetland community zones are distributed perpendicular to the Yellow River and adjacent to its pathway from the sea to the land (Yu et al., 2012). With the action of ocean dynamics, runoff, and sedimentation, the estuarine sediment continuously deposits, extends, swings, and diverts cyclically. The variables consist predominantly of fluctuations in dissolved oxygen, salinity, and sediment load within the water (Yu et al., 2012). Thus, the YRD has both extension and retrogradation, accompanied by rapid growth and degenerative succession of vegetation.

### Sampling

We chose five random sampling plots (0.5 m × 0.5 m, 25 m apart) (in wetlands formed since 1996), based on communities without (P1) or with vegetation dominated by *S. salsa* (P2), *T. chinensis* (P3), *P. australis* (P4), or *T. orientalis* (P5). The vegetation cover for P2, P3, P4, and P5 was 89, 83, 65, and 80%, respectively. Location and vegetation coverage information for each sampling plot are provided in Supplementary Figure S1 and Supplementary Table S1.

Three soil cores (0 to 20 cm depth) were taken from each of the five plots. Three subsamples from each soil core were selected randomly and homogenized as a composite sample for the plot. The composite sample was passed through a 2 mm sieve. Seasonal sampling was performed: July 2012 (summer), October 2012 (fall), February 2013 (winter), and May 2013 (spring). Thus, we collected 60 samples: 12 samples per plot per season. Each sample was placed in a sterile plastic bag, which was sealed and transported to the laboratory within 24 h after sampling. All samples were stored at -80°C for DNA extraction within 30 days and at 4°C for physicochemical measurements within 7 days.

### Physical and Chemical Characteristics

Soil water content (%), soil salinity (EC, μS cm^-1^), soil pH, inorganic ions (Ca^2+^, Mg^2+^, K^+^, Na^+^, Cl^-^, and SO_4_^2-^), total carbon (TC), and total nitrogen (TN) were determined and the detail methods can be found in our earlier work (Lv et al., 2016). Inorganic soil nitrogen content, including ammonium (NH_4_^+^), nitrate (NO_3_^-^), and nitrite (NO_2_^-^) was extracted from fresh soils with 2 M KCl and determined using an Astoria Analyzer 300 system (Lv et al., 2016). Soil organic matter (SOM) was determined using the K_2_CrO_7_ oxidation-colorimetric method (Walkley, 1947). All measurements were replicated three times for each sample (three subsamples were analyzed) and the average data were presented.

### DNA Extraction and Sequencing

Extraction of bacterial genomic DNA followed the same method as our previous work (Lv et al., 2016). The Ion Torrent (PGM) Platform was used for Microbial 16S rRNA Ion Tag and community metagenome sequencing; detailed information can be found in Whiteley et al. (2012) and Langille et al. (2013).

### Bioinformatic Analyses

The taxonomy of operational taxonomic units (OTUs) was classified with Greengene V. The relative abundance of each OTU was corrected by the estimated number of 16S rRNA gene copies and was normalized with the negative nominal model (McMurdie and Holmes, 2014), which minimized bias associated with sequencing coverage and allowed for comparison of results for all samples. Dominant OTUs were identified as OTUs with relative abundances greater than 0.1%. Based on these data, alpha-diversities, including OTU richness, Shannon’s index, Simpson index, and Shannon diversity index, were calculated with the R package vegan and detailed information can be found in Dixon (2003). Phylogenetic diversity (PD) was calculated with the R package picante (Kembel et al., 2010). Non-metric multidimensional scaling (NMDS) analyses were used for ordination based on the UniFrac phylogenetic distance matrix for bacterial community structure (OTUs) (Dixon, 2003). Analysis of similarity (ANOSIM) was employed to test differences between community groups (Dixon, 2003). Redundancy analysis (RDA) was used for determining relationships between bacterial community structures and environmental factors per Ma et al. (2018). Linear discriminant analysis effect size (LEfSe) method was used to discover taxonomic biomarkers (Segata et al., 2011). All statistical analyses and graphics were done using the R program (R Core Team, 2018). All statistical tests were considered significant at *P* < 0.05. These sequence data have been submitted to the SRA databases under accession number SRX1058187.

## Results

### Bacterial Community Composition in Newly Formed Wetlands

There were 158,693 reads identified from 60 soil samples, and the mean value of sequence number was 2715 (±1029) for each sample (Supplementary Figure S2). There were 6041 OTUs (at 97% sequence identity) identified from 60 soil samples. Most samples nearly attained the saturated stage, indicating that nearly all bacterial species were sequenced in our samples. Five samples coming from different sampling plots with small read numbers were not saturated and were removed from the analyses.

**FIGURE 1:**
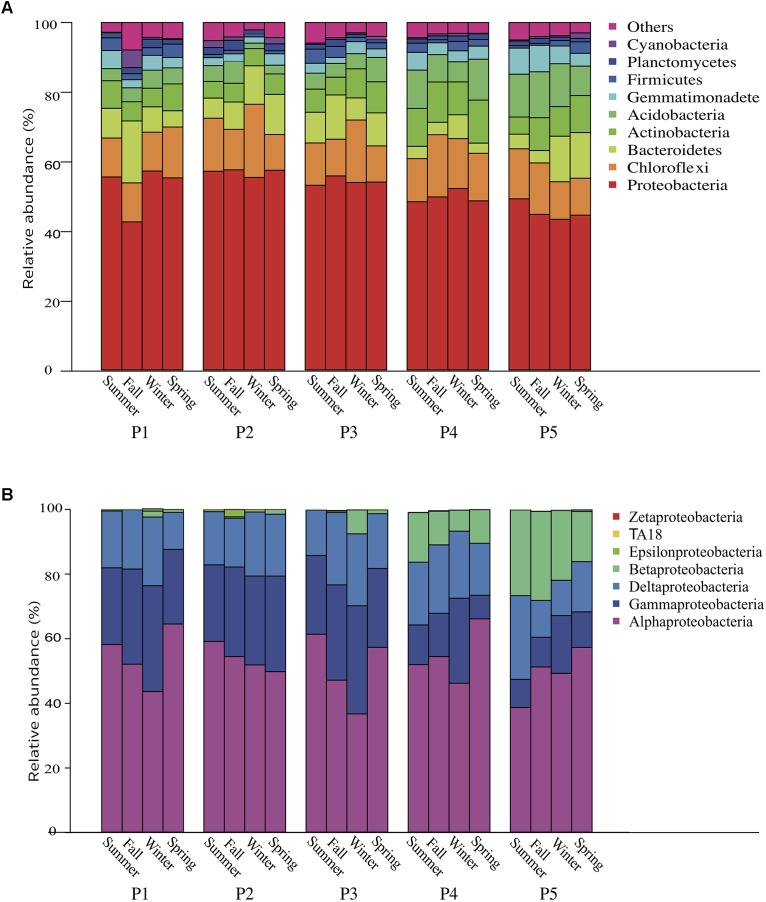
Relative abundance of **(A)** bacteria phyla and **(B)** Proteobacteria subphyla in the soil over four seasons in a newly formed wetland (P1, P2, P3, P4, and P5: plots without vegetation and vegetation dominated by *Suaeda salsa*, *Tamarix chinensis*, *Phragmites australis*, and *Typha orientalis*, respectively).

The bacterial communities were dominated by phyla Proteobacteria (51.8%) and Chloroflexi (13.3%), followed by Bacteroidetes (8.2%), Actinobacteria (7.7%), Acidobacteria (6.7%), Gemmatimonadetes (3.1%), Firmicutes (2.0%), Planctomycetes (1.5%), and Cyanobacteria (0.8%) (Figure 1). The dominant classes of Proteobacteria were Alphaproteobacteria (52.4%), Gammaproteobacteria (21.8%), Deltaproteobacteria (17.8%), and Betaproteobacteria (7.5%). From the saline bare land (P1) to Yellow River land (P5), the relative abundances of Cyanobacteria and Betaproteobacteria significantly decreased (*P* = 0.0479) and increased (*P* = 0.0146), respectively. There were significant differences among the four sampling seasons in Acidobacteria (*P* = 0.00169), Proteobacteria (*P* = 0.00925), Gemmatimonadetes (*P* = 0.001), Firmicutes (*P* = 0.0167), and Alphaproteobacteria (*P* = 0.0126).

**FIGURE 2:**
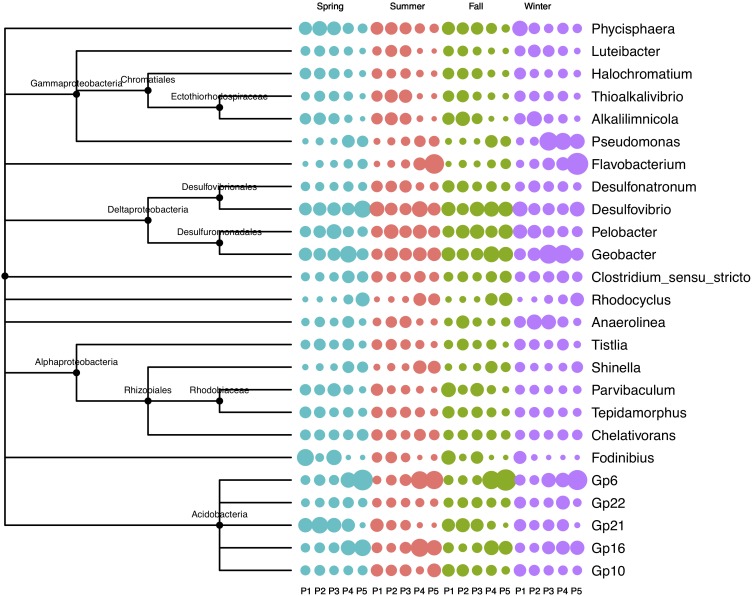
Relative abundance of soil bacterial community in four seasons in the newly formed wetland (P1, P2, P3, P4, and P5: plots without vegetation and vegetation dominated by *S. salsa*, *T. chinensis*, *P. australis*, and *T. orientalis*, respectively). Larger circles of the same color in the same row or the same column indicate greater relative abundance.

The five plots showed contrasting patterns in microbial composition: the relative abundance of *Fodinibius*, Gp21, *Alkalilimnicola*, *Phycisphaera*, and *Luteibacter* decreased gradually from P1 to P5, whereas that of *Geobacter*, *Rhodocyclus*, *Flavobacterium*, *Shinella*, *Pseudomonas*, Gp6, and Gp16 increased from P1 to P5 (Figure 2). In the transitional plot (P3), the most abundant OTUs were assigned to *Anaerolinea*, *Pseudomonas*, and *Geobacter* in winter, while no significant difference was found for the microbial composition in summer.

### Taxonomic Level of Bacterial Composition in Newly Formed Wetlands

**FIGURE 3:**
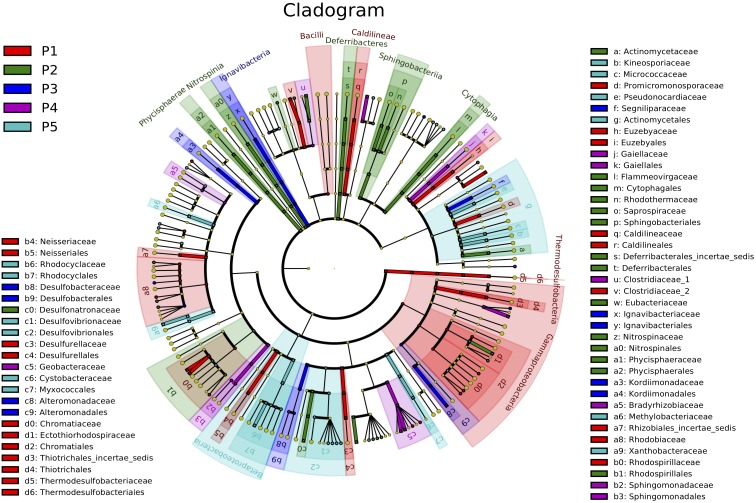
Bacteria biomarkers for the five plots showing taxonomic composition of soil bacterial community in newly formed wetlands (P1, P2, P3, P4, and P5: plots without vegetation and vegetation dominated by *S. salsa*, *T. chinensis*, *P. australis*, and *T. orientalis*, respectively).

To investigate taxonomic distribution, a statistical strategy was applied to discover taxonomic biomarkers by LEfSe (Xu et al., 2014). We identified 43 biomarker species (10 in P1, 11 in P2, 3 in P3, 5 in P4, and 14 in P5) that strongly differentiated soil using LEfSe software (Figure 3). Sets of different vegetation stage biomarkers were phylogenetically distinct at the order level. P1 and P2 were primarily associated with Gammaproteobacteria, while P4 and P5 were associated with Xanthobacteraceae and Chromatiales, and Thiotrichales, respectively. They were consistently more abundant as vegetation succeeded, as confirmed by biomarker abundance trajectories.

### Bacterial Community Diversity in Newly Formed Wetlands

**FIGURE 4:**
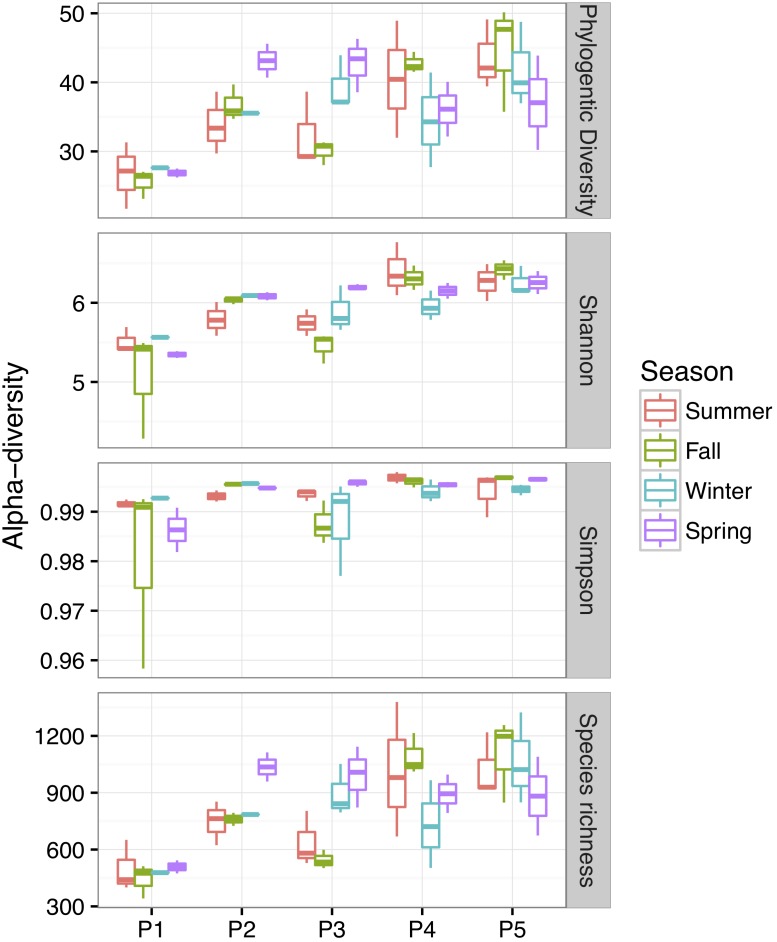
Alpha-diversity of the soil bacterial communities over four seasons in newly formed wetlands (P1, P2, P3, P4, and P5: plots without vegetation and vegetation dominated by *S. salsa*, *T. chinensis*, *P. australis*, and *T. orientalis*, respectively).

**FIGURE 5:**
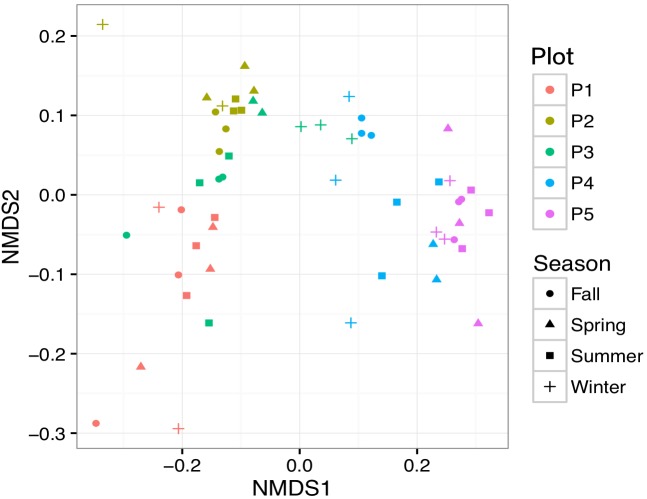
Non-metric multidimensional scaling plots for soil bacterial community structure based on UniFrac distance in newly formed wetlands. Samples from three plots are illustrated by different colors. Samples from four seasons are illustrated by different symbols. P1, P2, P3, P4, and P5: plots without vegetation and vegetation dominated by *S. salsa*, *T. chinensis*, *P. australis*, and *T. orientalis*, respectively.

Bacterial community alpha-diversity was significantly different among the four seasons (*P* < 0.01) (Figure 4). Alpha-diversity was lowest in P1 and increased from P2 to P5 (*P* < 0.01). Both the alpha-diversity index value and its rarefaction curves showed that P1 had the lowest diversity. To elucidate phylogenetic similarities, we employed the NMDS, based on Bray–Curtis and UniFrac distance, to assess the bacterial phylogenetic beta-diversity among the bacterial communities (Figure 5). Bacterial communities in the same plot were clustered together, and were dissimilar among the five plots (ANOSIM, *R*^2^ = 0.183, *P* = 0.001). The distances between bacterial communities represented the plot locations. However, the ANOSIM results did not show a significant difference in bacterial communities among seasons (*R*^2^ = 0.0096, *P* = 0.98), indicating that seasonal variation in temperature had little influence on bacterial community in newly formed wetlands in the YRD.

### Contribution of Environmental Factors to Bacterial Community Structure

**FIGURE 6:**
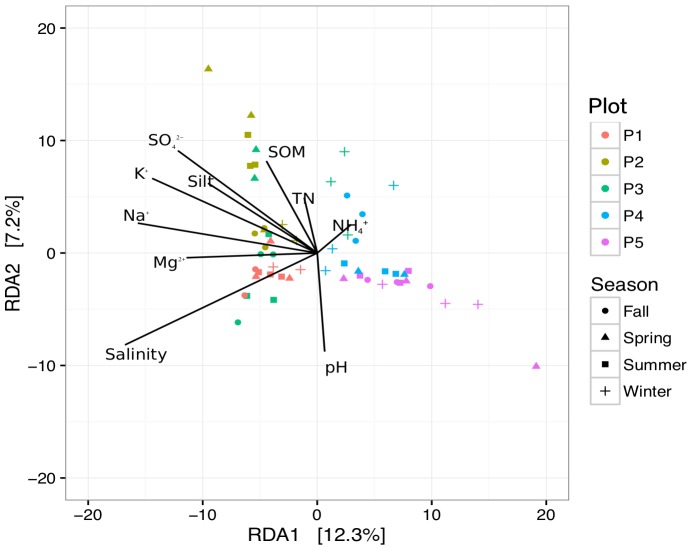
Redundancy analysis for sediments in newly formed wetlands, using bacterial community structure and environmental factors (P1, P2, P3, P4, and P5: plots without vegetation and vegetation dominated by *S. salsa*, *T. chinensis*, *P. australis*, and *T. orientalis*, respectively; SOM, soil organic matter; TN, total nitrogen).

According to our research results in the coastal wetland, environmental factors might play a significant role in bacterial community structure. To identify environmental factors that contribute to variations in bacterial community structure, we employed RDA to quantify environmental variables. In the first two axes, the RDA explained 17.5% of the variation, confirming clear separation of sites in terms of environmental factors (Figure 6). Axis 1 (RDA1) established a separation as vegetation succession progressed in the newly formed wetlands. Soil salinity was found to be a principal environmental factor affecting the bacterial community in the soil, as succession progressed (*R*^2^ = 0.175, *P* < 0.001). Soil salinity and the concentrations of Na^+^, K^+^, Mg^2+^, Ca^2+^, Cl^-^, and NO_3_^-^ deceased from P1 to P5, while there was no significant difference among the sampling seasons.

## Discussion

### Salinity Effect on Bacterial Community Composition

Soil salinity is recognized as a principle factor influencing bacterial community composition in newly formed, natural coastal wetlands in the YRD (Lv et al., 2016). High salinity influences coastal wetland ecosystems in several ways: suppressing plant growth and heterotrophic metabolism, decreasing soil quality and diversity of heterotrophic bacteria (e.g., Betaproteobacteria, Chloroflexi, and Bacteroidetes), and negatively impacting ecosystem stability (Abed et al., 2010; Székely et al., 2013; Morrissey et al., 2014; Xi et al., 2014; Yang et al., 2016). The difference in phylogenetic groups in wetlands with various types of vegetation could reflect the functional discrepancy of bacterial communities along a salinity gradient. The high abundance of halophilic bacteria in P1 and P2 (e.g., *Fodinibius*, *Alkalilimnicola*, *Phycisphaera*, and Gp21) were also common in marine and sediments (Fukunaga et al., 2009; Sorokin et al., 2010; Wang et al., 2012). Acidobacteria were reported frequently as dominant members in some mining environments (Teng et al., 2017). Its numerical dominance in P1 and P2 thus seemed to indicate its (potentially) important role in sulfur and iron cycling in these tailings. Compared with high salinity zones, the dominant genera (e.g., *Rhodocyclus*, *Flavobacterium*, and *Shinella*) were prevalent in the transition zone (P3). *Rhodocyclus* spp. live in aquatic habitats and prefer oligotrophic conditions; they perform anoxygenic photosynthesis under anoxic conditions (Zilles et al., 2002). In addition, high abundances of Flavobacteria, a well-known cold-adapted genera in marine ecosystems, were shown in P5 in winter (Abed et al., 2010; Georges et al., 2014; Raulf et al., 2015). The relative abundance of Cyanobacteria was higher than that reported in a previous study on a YRD tidal flat. In addition, the relative abundances of Cyanobacteria decreased significantly from the saline bare land (P1) to Yellow River shoal (P5). This may be explained by the increase of fine particles (clay and silt), which reduced their photosynthetic capacity and decreased their abundance (Al-Najjar et al., 2014). The relative abundance of Acidobacteria GP6 increased from the saline bare land (P1) to Yellow River shoal (P5) during all the sampling seasons. Previous studies reported that there was a positive relationship between the abundance of GP6 and the soil content of Ca, Mg, Mn, and B (Navarrete et al., 2013). Although we did not analyze the chemical properties, it might be a reasonable explanation for the present results since vegetation plays an important role in soil properties.

### Combined Effects of Temperature and Vegetation on Bacterial Community Diversity

Temperature has also been acknowledged as a principal factor influencing bacterial community diversity and function, which directly reflect seasonal temperature changes (Böer et al., 2009; Uroz et al., 2013). For instance, a significant difference in microbial community diversity and structure was found between winter and summer in Lake Erie sediments (Wilhelm et al., 2014). The lowest bacterial PD in winter was found in tidal flats, coastal water, and lake sediments (Patel et al., 2014; Wilhelm et al., 2014; Lv et al., 2016). The decrease in bacterial PD in winter is mainly because bacteria are in an inferior position in competition with fungi during low temperature (Schadt et al., 2003). In our previous study in the tidal flats of the YRD, we found that there was a decrease in the relative abundance of Actinobacteria, Alphaproteobacteria, and Anaerolineae, while there was an increase in Flavobacteriales in winter, compared with other seasons (Lv et al., 2016). However, no significant differences were found in bacterial communities among seasons, indicating that temperature had little influence on bacterial community in the newly formed wetlands. The reason for this phenomenon was unclear and needs to be investigated. Vegetation plays a significant role in coastal wetlands, since it can protect the seashore by attenuating waves, promoting sedimentation, preventing soil erosion, and influencing sedimentary dynamic processes of coastal zone tidal flat evolution (González-Alcaraz et al., 2015; Landi and Angiolini, 2015). During plant growth, plant roots help to form soil aggregate structure by promoting sedimentation and breaking up large soil particles (Liu et al., 2009; Yu et al., 2015b). Among the soil physical parameters measured in the salt marsh, clay and sand content are the major drivers influencing community composition (Diniandreote et al., 2014). In forest soil microbial communities, there was a strong positive relationship between clay content and functional gene diversity, abundance, and composition (Paula et al., 2014). Vegetation plays an important role in soil texture and influences the bacterial community indirectly. In newly formed coastal wetlands, vegetation increased from saline bare land (P1) to Yellow River shoal (P5), as soil salinity gradually decreased. Thus, as vegetation diversity increased from saline bare land (P1) to Yellow River shoal (P5), soil texture changed from coarse to fine particles (Yu et al., 2015b), and the bacterial diversity increased accordingly. Our results are consistent with earlier findings: vegetation coverage increased from 45 to 97% and succession progressed from saltwater vegetation to freshwater vegetation in the YRD (Yu et al., 2012).

### Combined Effects of Environmental Factors on Bacterial Community Structure

Environmental factors (e.g., soil pH, texture, nitrate, and nitrite) played a significant role in bacterial community composition and bacterial community diversity in a newly formed, natural coastal wetland. A consistent shift in community structure along a salinity gradient (from saline bare land to *T. orientalis* community) was mainly because of the combination of factors associated with coastal environment and plants. Although there was no tidal action effect, the saline bare land in the present study was characterized by late accumulation, short development period, and high heterogeneity of environment factors, which led to low bacterial community richness. Moreover, high salinity due to seawater invasion could decrease species richness. Therefore, as soil saline content decreased from saline bare land (P1) to the Yellow River shoal (P5), bacterial communities increased. Several researchers have revealed that, in coastal regions, salinity is one of the most important factors that affects microbial community structure (Székely et al., 2013; Vasquezcardenas et al., 2015; Lv et al., 2016). As discussed above, soil texture (soil particle size) has a more important effect on microbial community structure than soil pH and SOM, and a close relationship between soil microbial community and soil particle size was found (Sessitsch et al., 2001). Our results are consistent with these findings: soil particle size was an important environmental factor for soil bacterial community structure in newly formed wetlands. Nitrate and nitrite were recognized as major substrates for nitrifying and denitrifying bacteria, which play an important role in nitrification and denitrification (two important counter processes of biochemical cycling), especially in wetland ecosystems experiencing periodic drying and flooding (Choi et al., 2010; John et al., 2011; Peralta et al., 2013; Bai et al., 2014). Our results showed that there were positive correlations between Actinobacteria and Gemmatimonadetes and nitrite in freshwater wetlands (Zhang et al., 2014).

## Conclusion

In conclusion, bacterial community structure significantly shifted along a salinity gradient in newly formed coastal wetlands. RDA indicated that soil salinity exerted a significant impact on the microbial community in the sediment and explained 17.5% of the variability. Thus, there are still many unknowns and our understanding of microbial community diversity is still limited in wetland ecosystems. Our study demonstrated that vegetation altered bacterial community structure and function in newly formed, natural wetlands. These results provide new insights into the relationships between microbial communities and environmental conditions in newly formed wetlands, which are special and unique in coastal regions. Our study also offers a case study for understanding the structure and function change in bacterial communities under environmental changes in coastal ecosystems, and it provides a scientific basis for wetland ecosystem conservation and restoration.

## Author Contributions

XL, JY, and MD designed the project and revised the manuscript. JR, Y-BS, MY, and XL performed the experiment and data analysis. WL and XL wrote the manuscript.

## Conflict of Interest Statement

The authors declare that the research was conducted in the absence of any commercial or financial relationships that could be construed as a potential conflict of interest.

## Supplementary Material

FIGURE S1 Locations of plots in the Yellow River Delta (P1, P2, P3, P4, and P5: plots without vegetation and vegetation dominated by *Suaeda salsa*, *Tamarix chinensis*, *Phragmites australis*, and *Typha orientalis*, respectively).

FIGURE S2 Rarefaction curves for all samples.

TABLE S1 General condition of the sampling plots in the Yellow River Delta (P1, P2, P3, P4, and P5: plots without vegetation and vegetation dominated by *S. salsa*, *T. chinensis*, *P. australis*, and *T. orientalis*, respectively).
