# Supplementary figures and images for: Variations in Soil Bacterial Composition and Diversity in Newly Formed Coastal Wetlands
Source: Front Microbiol. 2019 Jan 9;9:3256. doi: 10.3389/fmicb.2018.03256 (PMC6333922; doi:10.3389/fmicb.2018.03256)

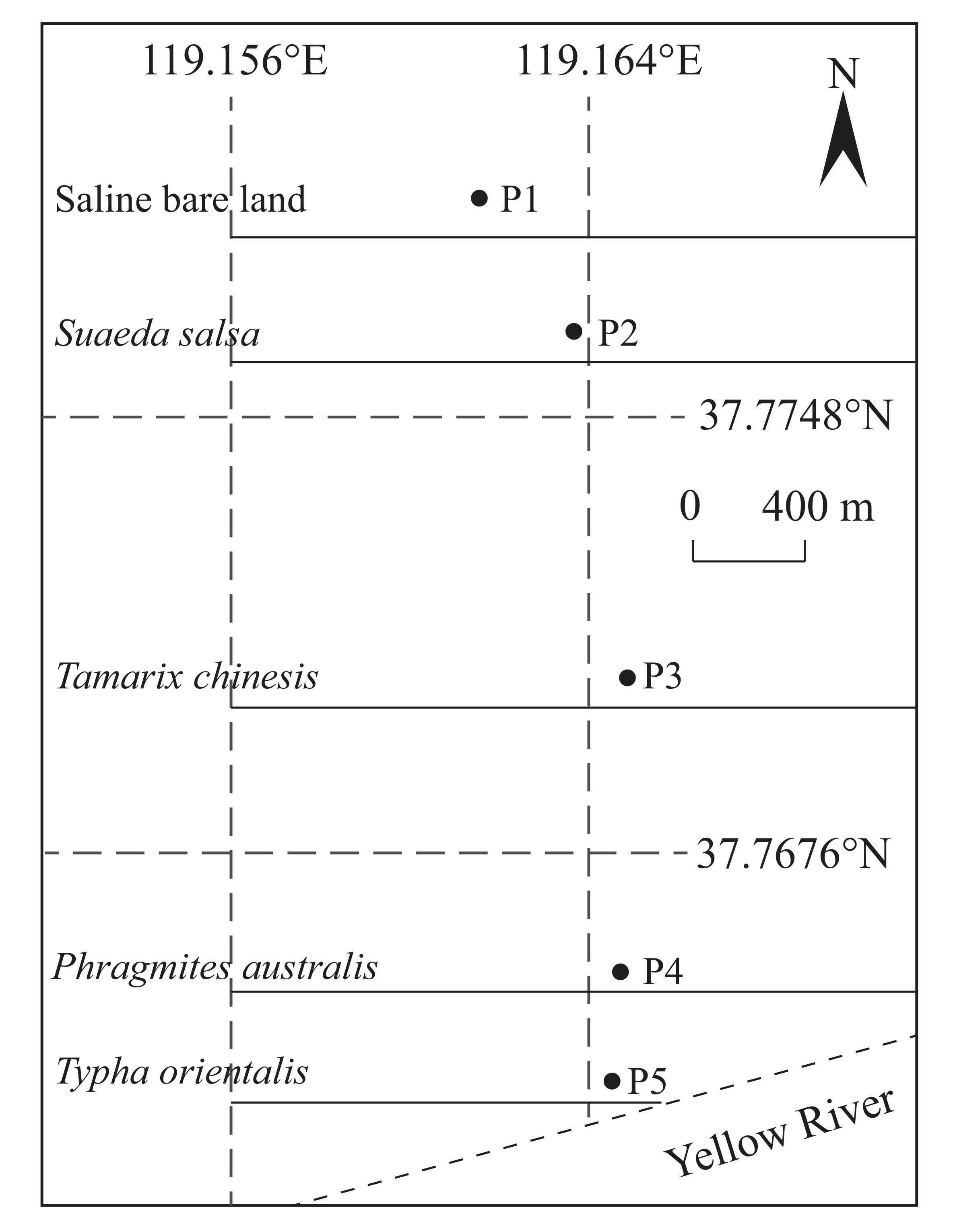

Supplement: FIGURE S1 — Locations of plots in the Yellow River Delta (P1, P2, P3, P4, and P5: plots without vegetation and vegetation dominated by Suaeda salsa, Tamarix chinensis, Phragmites australis, and Typha orientalis, respectively). [file Image_1.JPEG]

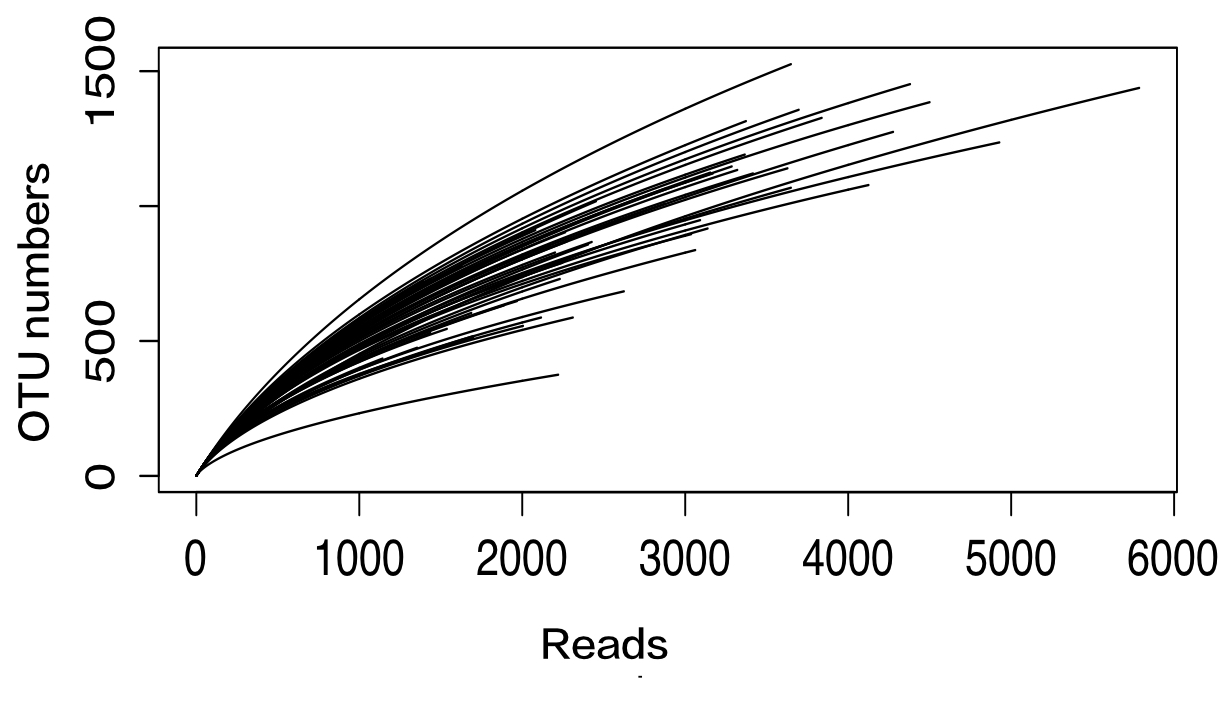

Supplement: FIGURE S2 — Rarefaction curves for all samples. [file Image_2.JPEG]
