# Supplementary material for: Variations in Soil Bacterial Composition and Diversity in Newly Formed Coastal Wetlands
Source: Front Microbiol. 2019 Jan 9;9:3256. doi: 10.3389/fmicb.2018.03256 (PMC6333922; doi:10.3389/fmicb.2018.03256)
Supplement: TABLE S1 — General condition of the sampling plots in the Yellow River Delta (P1, P2, P3, P4, and P5: plots without vegetation and vegetation dominated by S. salsa, T. chinensis, P. australis, and T. orientalis, respectively). [file Table_1.doc]

Supplementary Table 1 General condition of the sampling plots in the Yellow River Delta (P1, P2, P3, P4, and P5: plots without vegetation and vegetation dominated by *Suaeda salsa*, *Tamarix chinesis*, *Phragmites australis*, and *Typha orientalis*, respectively)

| Sampling plot | Vegetation cover (%) | Plant species |
| --- | --- | --- |
| P1 | - | - |
| P2 | 89 | *Suaeda salsa* |
| P3 | 83 | *Suaeda salsa*  *Tamarix chinesis* |
| P4 | 65 | *Phragmites australis*  *Cynanchum chinense* |
| P5 | 80 | *Typha orientalis*  *Phragmites australis*  *Calamagrostis seudophragmites*  *Triarrhena sacchariflora Chrysopogon aciculatus Apocynum venetum*  *Cirsium japonicum Fisch*  *Suaeda salsa* |
